# Depression and Anxiety in the Saudi Population: Epidemiological Profiles from Health Surveys and Mental Health Services

**DOI:** 10.1177/00469580251382027

**Published:** 2025-10-16

**Authors:** Ahmed Yahya Almakrob, Ahmed Alduais

**Affiliations:** 1 Department of English Language and Literature, College of Sciences and Humanities, Prince Sattam Bin Abdulaziz University, Al-Kharj, Saudi Arabia; 2 Department of Psychology, Norwegian University of Science and Technology, Trondheim, Norway

**Keywords:** mental health, depression, anxiety, good health and wellbeing, quality education

## Abstract

Depression and anxiety are prominent contributors to the global disease burden, with significant health system implications particularly in underexamined contexts such as Saudi Arabia. While existing studies often target discrete subpopulations, few have synthesized national data to evaluate mental health prevalence alongside service utilization. This study addresses that gap by analyzing 4 national datasets—the 2024 National Health Survey, the 2024 Woman and Child Health Survey, the 2017 Disability Survey, and the Ministry of Health’s mental health reports—using a framework grounded in WHO standards (ie, the Mental Health Action Plan). We operationalized 8 analytical indicators covering prevalence, symptom frequency, treatment usage, diagnostic distribution, comorbidity patterns, and health system responsiveness. Descriptive statistics and latent class analyses revealed consistently high prevalence of depression and anxiety among adults, children, individuals with disabilities, and healthcare users. Women, adolescents, and chronically ill individuals reported higher symptom severity and lower access to tailored interventions. Mental health service data emphasized diagnostic overrepresentation of psychotic disorders and under-documentation of emotional distress, indicating a systemic diagnostic skew. The findings expose critical gaps between population mental health needs and current diagnostic-service frameworks. By informing SDG 3 (promoting mental health and well-being) and SDG 4 (inclusive education and well-being for children), this study supports enhanced policy design for early identification, equity-focused care, and integration of functional assessment within Saudi Arabia’s mental health system.

**Highlights**

● This study integrates four national-level datasets to provide the first triangulated epidemiological profile of depression and anxiety in Saudi Arabia.

● Findings reveal sex, age, and disability-related disparities, with adolescents, women, and individuals with disabilities showing higher symptom burdens.

● Clinical service data show a diagnostic skew, with schizophrenia and mood disorders dominating, while common conditions like anxiety are underrepresented.

● Chi-square and clustering analyses highlight systemic gaps, including under-detection of mild and pediatric cases, and mismatches between community prevalence and service provision.

● Results inform Vision 2030 reforms, emphasizing the need for early screening, community-based services, and culturally adapted interventions.

## Introduction

### Prevalence of Depression and Anxiety Internationally

Depression and anxiety are the most prevalent mental health disorders globally, affecting populations across diverse cultural and socioeconomic contexts. The point prevalence of depression is estimated at 3.8%, with higher rates among adults (5.0%) and older adults over 60 (5.7%).^1^ Estimates vary substantially: the 12-month prevalence of major depressive disorder ranges from 0.3% to over 10.2% depending on region, healthcare access, and diagnostic tools.^2^ In Europe, lifetime prevalence is about 13% and 1-year prevalence 4%, while Poland reports depressive episodes in roughly 3% of adults.^3,4^ According to the Global Burden of Disease Study, depression prevalence rose globally between 1990 and 2019, particularly among working-age adults.^5^ Disparities are pronounced: women, elderly groups, and socioeconomically disadvantaged populations consistently report higher prevalence.^3,6^

Anxiety disorders are even more widespread, with lifetime prevalence in the U.S. reaching 28.8% and 12-month prevalence at 18.1%.^7^ Europe shows annual prevalence around 14%, though rates differ globally—from 2.1% in East Asia to 6.1% in North Africa/Middle East.^8^ Anxiety is more common in women and young people, especially those aged 10 to 24.^5,9^ The overlap between disorders is striking: nearly 87% of individuals with depression also experience anxiety symptoms, complicating both diagnosis and treatment.^10^

Global patterns reflect multiple risk factors including female gender, low socioeconomic status, stress exposure, and stigma.^2,11^ National prevalence is further shaped by sociodemographic indices such as income inequality, rurality, and aging.^12^ Cultural dimensions also matter: Schwartz’s values of egalitarianism and hierarchy correlate with national rates of mental disorders.^13^ The COVID-19 pandemic intensified the burden, with increased symptoms among vulnerable groups such as cancer patients^14^ and international students.^9^ In response, health systems are testing scalable interventions, including culturally adapted psychotherapies and AI-based diagnostic supports.^10,15^ Despite these innovations, inequities in access and gaps in population-level surveillance persist, underscoring the need for stronger global monitoring and context-sensitive strategies.

### Depression and Anxiety in the DSM-5-TR, ICD-11, and ICF

The classification of depression and anxiety has been refined through 3 major frameworks: the DSM-5-TR, ICD-11, and ICF. The DSM-5-TR outlines criteria for disorders such as major depressive disorder, persistent depressive disorder, and disruptive mood dysregulation disorder, emphasizing persistent sadness, cognitive impairment, and somatic complaints that impair functioning.^16^ Anxiety disorders are categorized separately into generalized anxiety disorder, panic disorder, social anxiety disorder, and specific phobias, with obsessive-compulsive and trauma-related disorders now placed in distinct chapters to reflect evolving conceptual boundaries.^17,18^ Importantly, DSM-5-TR incorporates cultural perspectives, recognizing that symptom interpretation may vary across societies, a critical consideration in global mental health.^18^

The ICD-11, developed by the WHO, advances classification with greater clinical flexibility and global applicability. Among its innovations is the recognition of “anxious depression,” defined by co-occurring depressive and anxiety symptoms persisting for at least 2 weeks, a diagnosis especially useful in primary care.^19,20^ ICD-11 also redefines anxiety and fear-related disorders by eliminating hierarchical rules, grouping conditions by predominant symptoms of apprehension and fear.^21^ Disorders such as separation anxiety and selective mutism, once restricted to childhood, are now extended across the lifespan.^22^ These adjustments reduce diagnostic ambiguity and encourage earlier intervention in diverse health settings.^19^

The ICF adds a functional perspective, evaluating how depression and anxiety affect activities, participation, and interactions with the environment. Unlike DSM-5 or ICD-11, the ICF is not diagnostic but situates mental health within a biopsychosocial model, focusing on capacity, performance, and barriers to social engagement.^23^ It highlights the impact of symptoms on employment, social participation, and healthcare access, complementing diagnostic tools by linking disorders to lived functioning. The ICF framework also aligns with DSM-5′s categorical approach and ICD-11’s dimensional model by recognizing comorbidity and promoting holistic care planning.^24-26^ Together, DSM-5-TR, ICD-11, and ICF provide complementary diagnostic, epidemiological, and functional lenses that strengthen understanding and management of depression and anxiety across cultural and clinical contexts.

### Depression and Anxiety in Saudi Arabia

Depression and anxiety are growing public health challenges in Saudi Arabia, with a notable burden across multiple demographic groups. National screening data revealed that 12.7% and 12.4% of the Saudi population are at risk for major depressive disorder (MDD) and generalized anxiety disorder (GAD), respectively, highlighting a substantial proportion of individuals living with undiagnosed or untreated mental health issues.^27^ These findings were exacerbated during the COVID-19 pandemic, when 17.1% of the general population reported moderate to severe depressive symptoms, and 10% reported moderate to severe anxiety symptoms.^28^ Several risk factors have been identified, including female gender, chronic physical illness, and younger age, particularly among those exposed to stressful life events or lifestyle vulnerabilities such as smoking and lack of physical activity.^27,28^

The prevalence is particularly high among specific subgroups such as healthcare workers and university students. In a study assessing the psychological impact of COVID-19 on healthcare professionals, depression and anxiety rates reached 54.69% and 60.88%, respectively.^29^ Nurses and those with pre-existing chronic conditions were especially vulnerable.^29^ Similarly, alarming rates were documented among university students, with 81.5% reporting depressive symptoms and 63.6% experiencing anxiety in 1 Jeddah-based study.^30^ Another survey among King Abdulaziz University freshmen reported 80.4% and 71.8% rates for depression and anxiety, respectively, driven largely by academic stress, chronic illness, and poor coping mechanisms.^31^ These findings emphasize that students and frontline healthcare providers are 2 of the most psychologically affected populations in the Kingdom.

Mental health concerns also extend to pregnant women, caregivers, and working women, further illustrating the widespread impact of depression and anxiety in Saudi society. Among pregnant women in the Jazan region, 62.6% experienced depression and 68.7% experienced anxiety during pregnancy.^32^ Similarly, caregivers of hospitalized patients reported striking rates of psychological distress, with 72.8% suffering from depression and 76.5% from anxiety.^33^ Among working women, nearly half reported mild to moderate levels of both depression and anxiety, often associated with psychosocial stressors and perinatal issues.^34^ Despite these high prevalence rates, barriers to mental health care remain, underscoring the urgent need for regular screenings and culturally sensitive interventions aimed at early detection, prevention, and support services for these at-risk populations.^27,35^

The Saudi healthcare system differs structurally from other MENA countries through its mixed economy model, where the Ministry of Health (MOH) provides around 60% of healthcare services, complemented by other governmental agencies and a growing private sector.^29,36^ Unlike several MENA states where access to care is uneven or heavily reliant on out-of-pocket expenditure, Saudi Arabia guarantees free comprehensive healthcare to its citizens, funded primarily by state revenues from oil.^37,38^ However, the system faces challenges such as workforce shortages, rising costs, and increasing demand due to demographic and epidemiological transitions.^39^ Within mental health specifically, Saudi Arabia has taken notable steps toward reform. The National Mental Health Policy was established in 2006 to integrate mental health into primary care, followed by the Mental Health Law of 2014, which aligned with WHO standards, albeit with limited mechanisms for safeguarding patient rights.^40,41^ More recently, initiatives such as the Wazen performance program and e-mental health services aim to enhance service delivery, while psychotherapy remains underdeveloped and shaped by cultural considerations.^42,43^ These developments position Saudi Arabia as a unique case in the region, where systemic modernization intersects with longstanding cultural and structural barriers to mental healthcare.

### Purpose of the Present Study

Although global and regional evidence consistently underscores the high prevalence and impact of depression and anxiety, much of the existing research in Saudi Arabia remains limited in scope, focusing on specific groups such as university students, healthcare workers, or pregnant women.^29,31,32^ These fragmented studies often lack integration across datasets and provide little insight into how population-level screenings align with service delivery patterns. This study addresses this gap by triangulating data from the 2024 National Health Survey, the 2024 Woman and Child Health Survey, the 2017 Disability Survey, and mental health service records from the Ministry of Health.

The analytical approach is grounded in the frameworks established by the World Health Organization (WHO), particularly the ICD-11 diagnostic system, the ICF, and the WHO Mental Health Action Plan, which emphasize early detection, functional assessment, and service integration. Furthermore, this study contributes to the advancement of the United Nations Sustainable Development Goals (SDGs), particularly SDG 3.4, which aims to reduce premature mortality from non-communicable diseases, including mental health disorders, through prevention and treatment, and to promote mental well-being.^44^

Guided by this framework, the study addresses 6 research questions examining prevalence patterns by age and sex, symptom frequency in children and individuals with disabilities, diagnostic trends in mental health services, and latent population clusters. It also investigates how convergent insights from survey and service-based data can illuminate pathways for earlier diagnosis and improved healthcare interventions for depression and anxiety in Saudi Arabia.

### Research Questions

1. What are the sex- and age-related patterns in the prevalence of depression and anxiety among individuals aged 15 years and above in Saudi Arabia, and how do these vary in terms of symptom severity?
2. (*Covers Indicators 1-4: prevalence by sex and age for both depression and anxiety, from the National Health Survey 2024*)
3. To what extent do children aged 11 to 14 in Saudi Arabia exhibit symptoms of depression and anxiety, and how do these presentations differ by sex and age subgroup?
4. (*Covers Indicators 5 and 6: prevalence in children, from the Woman and Child Health Survey 2024*)
5. How do self-reported frequencies of anxiety and depression symptoms and treatment usage differ by sex among individuals with disabilities aged 15 and above in Saudi Arabia?
6. (*Covers Indicator 7: frequency of symptoms and treatment in the Disability Survey 2017*)
7. What are the dominant diagnostic categories of mental and behavioral disorders recorded in Ministry of Health mental health departments, and how do outpatient and inpatient visit patterns reflect service utilization across diagnostic groups?
8. (*Covers Indicator 8: ICD-10-based disorder groups from the MOH mental health services report*)
9. How do symptom severity and co-occurrence of depression and anxiety vary across age and sex groups, and what clusters can be identified in population-level mental health profiles?
10. (*Synthesizes data from Indicators 1 to 6; allows for clustering analysis*)
11. What do the converging insights from survey-based prevalence data and clinical service records suggest about gaps and opportunities in national-level mental health screening, diagnosis, and early intervention strategies in Saudi Arabia?
12. (*Integrative synthesis across all indicators, linking surveillance and health systems*)

## Methods

### Sample

This study relied on nationally representative datasets derived from 3 official health surveillance instruments conducted in Saudi Arabia: the National Health Survey 2024, the Woman and Child Health Survey 2024, and the Disability Survey 2017. All surveys were administered by the General Authority for Statistics (GASTAT) using stratified multistage probability sampling to ensure representativeness across Saudi Arabia’s 13 administrative regions and 151 affiliated governorates.^45^ The sampling design was constructed to proportionally reflect both Saudi and non-Saudi residents living in private households. The National Health Survey and Woman and Child Health Survey targeted individuals aged 15 and older and children aged 11 to 14, respectively, and captured sex-, age-, and region-disaggregated data related to depression and anxiety using standardized self-report instruments.^45^

The surveys employed a hierarchical sample framework with primary sampling units (enumeration areas) selected from a national master sampling frame, followed by household selection using systematic random sampling. Individual-level data were collected via face-to-face interviews conducted by trained field personnel using tablets with structured digital questionnaires on June 26, 2024.^46^ Classification codes such as ISIC4 and ISCO_08 were integrated into survey design to ensure international comparability and diagnostic validity of social and health indicators. See Supplemental File 1 for the Washington Group Extended Set on Functioning.

In parallel, the Disability Survey 2017 provided a purposive subset focusing on individuals aged 15 years and older who reported 1 or more disabilities. The survey applied a similar sampling architecture to maximize regional and demographic inclusivity while targeting physical, cognitive, and psychosocial impairments.^47^ This dataset enabled focused analysis of depression and anxiety frequency, and medication use among persons with disabilities. The combined use of these survey platforms allowed for robust population-level estimates of mental health conditions disaggregated by age, sex, and disability status.

### Design

This study employed a cross-sectional, multi-source epidemiological design aimed at characterizing national-level patterns of depression and anxiety prevalence, service utilization, and subgroup disparities in Saudi Arabia. Drawing on secondary data from 3 independently administered national datasets—the National Health Survey 2024, the Woman and Child Health Survey 2024, and the Disability Survey 2017—as well as administrative clinical service records from the Ministry of Health’s 2018 mental health reports, the study applied a stratified population health approach. Each data source represented a distinct surveillance layer: self-reported mental health symptoms in the general population, childhood-specific emotional health indicators, and condition-specific service interactions in clinical settings. The integration of these data enabled the construction of an epidemiological profile that accounts for both community-based mental health burden and institutional service responses, a design particularly suited for identifying gaps in mental health policy, screening, and intervention strategies.^45,48^

The structure of the study followed a theory-informed analytic sequence, anchored in the WHO Mental Health Action Plan framework.^49^ Six research questions guided the analysis, each mapped to specific indicators extracted from the data sources. These questions addressed (1) age- and sex-based prevalence of depression and anxiety, (2) pediatric patterns, (3) frequency and treatment burden among individuals with disabilities, (4) utilization of clinical services by ICD-10 diagnostic groups, (5) symptom co-occurrence and latent mental health clustering, and (6) integration of surveillance and service delivery data. This layered design allowed for both disaggregated analysis (eg, age-by-sex breakdowns) and holistic synthesis (eg, integrative system-level interpretation). The cross-sectional nature of all datasets ensured temporal alignment, with major surveys conducted contemporaneously in 2024 and the clinical data reflecting the most recent available figures. The study design aligns with internationally recommended practices for secondary epidemiological research using administrative and survey-linked mental health data.^50,51^ This study is reported in accordance with the STROBE (Strengthening the Reporting of Observational Studies in Epidemiology) checklist for cross-sectional studies (See Supplemental File 2.).^52-54^

### Measures

This study employed 8 mental health indicators across 4 data platforms to construct a multidimensional assessment of depression and anxiety in Saudi Arabia. Indicators 1 through 4 were drawn from the National Health Survey 2024 and measured self-reported symptoms of depression and anxiety among individuals aged 15 years and older. These were stratified by age group and sex, and presented both mild and moderate/severe symptom categories as per the survey’s coding schema, which was based on the ICF and WHO mental health screening instruments. Indicators 5 and 6, derived from the Woman and Child Health Survey 2024, extended this analysis to children aged 11 to 14, capturing emotional disorders and nervousness by sex and age subgroup using child-appropriate proxies and caregiver report mechanisms. Indicator 7 from the Disability Survey 2017 focused on individuals aged 15+ with disabilities, using structured modules to assess the frequency of anxiety and depression and the regularity of related medication usage. Finally, Indicator 8, from the Ministry of Health Mental Health Reports (2018), provided administrative data on both outpatient and inpatient visits to psychiatric departments categorized using ICD-10 mental and behavioral disorder groups (eg, F30-F39, F40-F48).

Depression and anxiety were assessed using the Washington Group Extended Set on Functioning (WG-ES), which is widely adopted for population-based surveys and grounded in the WHO’s International Classification of Functioning, Disability and Health (ICF).^55^ The WG-ES affect module includes 6 standardized items: 3 on anxiety (ANX_1-ANX_3) and 3 on depression (DEP_1-DEP_3). Respondents were asked about frequency of symptoms (daily, weekly, monthly, a few times a year, or never), whether they used medication for these feelings, and their perceived severity (a little, a lot, or somewhere in between). For analysis, we classified individuals as having mild symptoms if they reported “a little” on severity with monthly or less frequent occurrence; moderate symptoms if they reported “a lot” or “somewhere in between” with weekly frequency; and severe symptoms if they reported daily occurrence with severity ratings of “a lot” or concurrent medication use. These operational criteria ensured consistency across datasets while aligning with international survey practices.

To ensure internal coherence across these data sources, the study utilized a cross-source triangulation approach. First, quantitative prevalence data from nationally representative surveys (Indicators 1-6) were used to estimate population-level symptom distributions across sex and age categories. Second, clinical service data (Indicator 8) were analyzed to evaluate diagnostic concentration and service utilization trends, disaggregated by ICD-10 disorder groups. Third, subpopulation-specific data (children and individuals with disabilities; Indicators 5-7) allowed for deep-dives into underserved and potentially at-risk demographic groups. This triangulation approach enabled both vertical integration (linking symptom experience to treatment access) and horizontal integration (comparing different population groups). It also ensured that both survey-based and health system data were contextualized within a unified measurement logic—supporting interpretive consistency and facilitating system-level conclusions.^51^

All indicators were imported into a unified analytical dataset using structured metadata, cleaned, recoded, and analyzed using Python 3.11 and Pandas. Visual analytics were generated with Matplotlib and Seaborn libraries. Clustering analyses were based on z-score normalized prevalence rates to identify latent groupings using unsupervised machine learning (*k*-means algorithm), allowing for identification of mental health profiles across age groups. Each indicator was mapped to corresponding WHO Mental Health Action Plan domains to support policy relevance. All available cases within each dataset were included in the analysis, as the scope of depression- and anxiety-related variables was limited. This comprehensive inclusion strategy was intentional to maximize representativeness and comparability across the 4 national sources. No exclusion criteria were applied beyond the inherent design of the surveys. Moreover, the accessed databases did not contain missing entries for the depression- and anxiety-specific items used in this study, which ensured complete case analyses without imputation procedures.

## Procedure

### Data Collection

Data for this study were drawn from secondary sources publicly released by national institutions in Saudi Arabia, specifically the GASTAT and the Ministry of Health (MOH). Collection methods varied by source but maintained consistent principles of rigor, standardization, and national representativeness. The National Health Survey 2024 and the Woman and Child Health Survey 2024 were both administered by GASTAT through structured, face-to-face interviews conducted in private households using electronic tablets. The surveys collected self-reported mental health data from adults (15+) and children (11-14) respectively, and adhered to a national reference day (June 26, 2024) for consistent temporal alignment. The Disability Survey 2017 similarly used household enumeration techniques and included disability-specific mental health modules. For administrative health records, the study accessed MOH’s 2018 mental health reports, which compiled outpatient and inpatient data coded by ICD-10 groups from all MOH-affiliated psychiatric departments. All data sources are publicly available through Saudi Arabia’s Open Data Portal, ensuring transparency, replicability, and ethical compliance with public-use datasets.^56^ See Table 1 for detailed list of all data used.

**Table 1.** Collected Data and Used Indicators.

Indicator No.	Title	Source
1	Prevalence of depression among population (15 years and above) by sex and age	National Health Survey 2024
2	Prevalence of depression among population (15 years and above) by sex and region	National Health Survey 2024
3	Prevalence of anxiety among population (15 years and above) by sex and age	National Health Survey 2024
4	Prevalence of anxiety among population (15 years and above) by sex and region	National Health Survey 2024
5	Prevalence of depression among children (11-14 years) by sex and age	Woman and Child Health Survey 2024
6	Prevalence of anxiety among children (11-14 years) by sex and age	Woman and Child Health Survey 2024
7	Saudi population with disability (15 years and over) by sex and frequency of anxiety and depression and medication taken	Disability Survey 2017
8	Outpatients and inpatients in mental health departments (MOH) by main ICD-10 mental disorder groups (2018G)	Ministry of Health – Mental Health Reports (2018)

Although the Disability Survey was conducted in 2017, it was included in the present analysis because it remains the most recent nationally representative dataset specifically addressing individuals with disabilities in Saudi Arabia. This subgroup is typically underrepresented in population health surveys, and their inclusion provides critical insight into equity-related dimensions of depression and anxiety. We acknowledge the temporal gap compared to the 2024 surveys and consider this a limitation; however, the dataset offers unique value by capturing disability-specific prevalence and treatment patterns unavailable elsewhere.

### Data Analysis

The analytical framework was both descriptive and inferential, involving univariate and multivariate techniques. Initial analyses included frequency distributions and prevalence estimation disaggregated by sex, age, and disability status. These were followed by visualization techniques (eg, bar charts, heatmaps, clustering plots) to identify patterns across indicators. A key component of the analysis was cross-source triangulation: quantitative symptom prevalence data (Indicators 1-6), subgroup-specific data (children and individuals with disabilities; Indicators 5-7), and clinical service records (Indicator 8) were merged conceptually to assess population burden versus institutional response. *K*-means clustering was used to uncover latent population profiles of co-occurring symptoms. All data were managed and analyzed using Python 3.11, employing Pandas for data wrangling, Matplotlib and Seaborn for visualizations, and Scikit-learn for clustering algorithms. This multimethod approach enabled system-level interpretation and facilitated alignment with global mental health governance frameworks.

### Ethical Considerations

The study is based entirely on de-identified, aggregate-level secondary data obtained from public datasets, thus exempting it from formal institutional ethics review under most national and international research governance standards.^57^ None of the datasets used contain personally identifiable information or involve direct interaction with human participants. All data were accessed through official and publicly sanctioned channels including the Saudi Open Data Portal, GASTAT’s website, and the MOH’s public archives. The study conforms to the principles outlined in the Declaration of Helsinki regarding the ethical use of secondary health data.^57^ In reporting results, all interpretations are contextualized to avoid overgeneralization or misrepresentation of vulnerable groups, and results are presented in aggregated form to preserve confidentiality.

## Results

**Figure 1.**
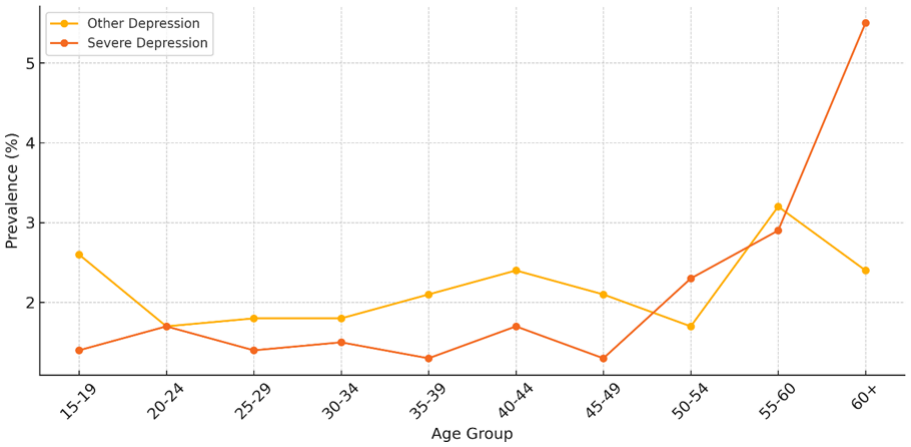
Depression prevalence by age group.

Figure 1 illustrates the prevalence of “other” and “severe” forms of depression among individuals aged 15 and above in Saudi Arabia, as derived from the National Health Survey 2024. Notably, the figure indicates a clear upward trend in severe depression with increasing age, rising sharply in those aged 55 and above, peaking at 5.5% among individuals over 60 years old. Conversely, “other” forms of depression remain relatively stable across age groups, with slight peaks in the 15 to 19 and 55 to 60 age brackets. These findings suggest age is a significant determinant in the severity of depression, underscoring the importance of age-targeted mental health interventions and screening protocols in older populations.

**Figure 2.**
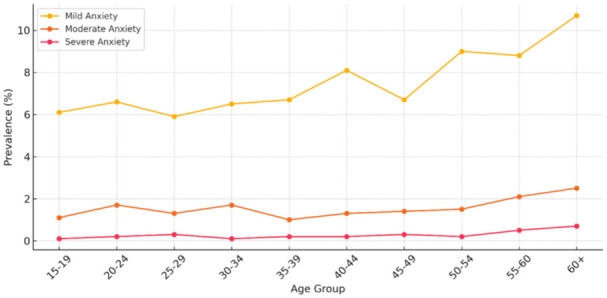
Anxiety prevalence by age group.

Figure 2 presents the distribution of mild, moderate, and severe anxiety symptoms across different age groups based on self-reported data from the National Health Survey 2024. A progressive increase in anxiety severity is evident with advancing age, with mild anxiety rising from 6.1% among those aged 15 to 19 to 10.7% in the 60+ age group. Similarly, moderate and severe anxiety show upward trends, albeit more gradual, with severe anxiety increasing sevenfold from 0.1% to 0.7%. These results highlight a critical need for proactive mental health strategies focused on aging populations, particularly considering the compounding effects of both moderate and severe symptoms in older adults.

**Figure 3.**
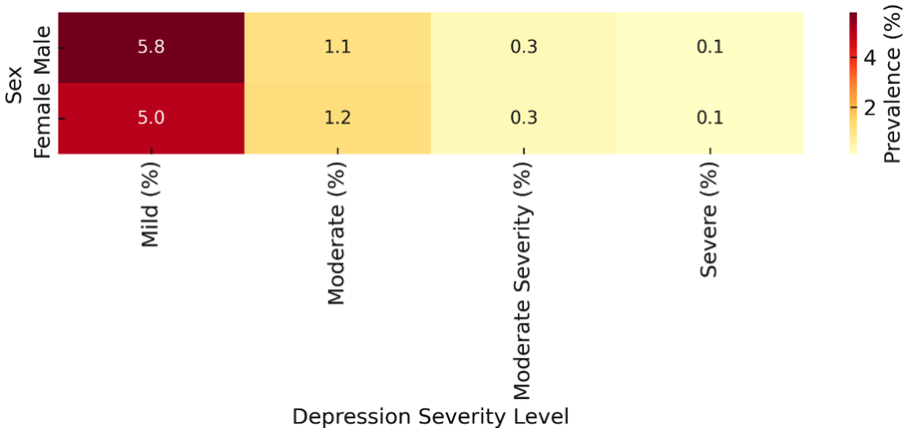
Heatmap of depression severity in children (11-14) by sex.

Figure 3 presents a heatmap visualization of self-reported depression symptoms among Saudi children aged 11 to 14, categorized by sex and severity. Mild depression is the most commonly reported category, affecting 5.8% of boys and 5.0% of girls. Moderate depression shows near-equal distribution (1.1% in boys, 1.2% in girls), while moderate severity and severe depression each affect only 0.3% and 0.1%, respectively, across both sexes. These findings indicate that while mild symptoms are relatively common and slightly more pronounced in boys, more severe forms of depression are rare and evenly distributed across sexes. This pattern suggests the early onset of depressive symptoms may not yet strongly diverge by sex, reinforcing the importance of inclusive early screening strategies that can detect mild symptoms before escalation.

**Figure 4.**
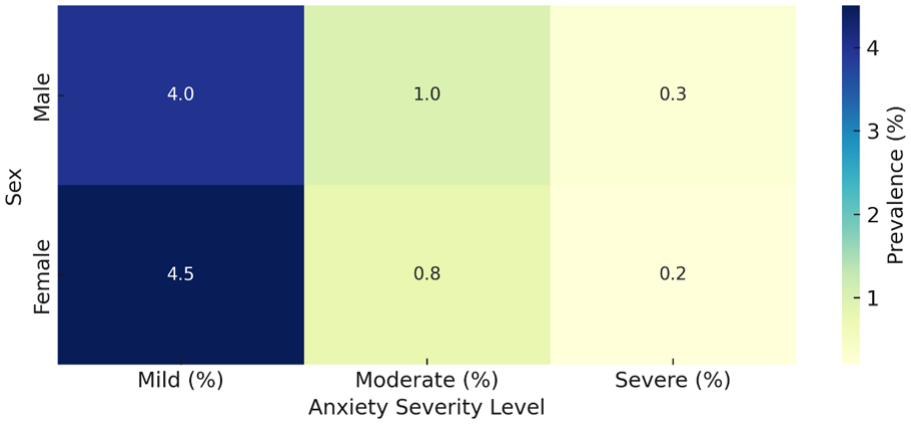
Heatmap of anxiety severity in children (11-14) by sex.

Figure 4 illustrates the prevalence of anxiety symptoms among children aged 11 to 14, differentiated by sex and severity level. Mild anxiety is slightly more prevalent among girls (4.5%) compared to boys (4.0%), suggesting early perceptual or social stress differentials. Conversely, boys report higher levels of both moderate (1.0%) and severe anxiety (0.3%) compared to girls (0.8% and 0.2%, respectively). The concentration of anxiety symptoms in the mild category, along with the slight male predominance in more severe forms, indicates a potential vulnerability in boys for symptom escalation. These data underscore the importance of early intervention efforts that are sensitive to subtle but important sex-based differences in anxiety expression during early adolescence.

**Figure 5.**
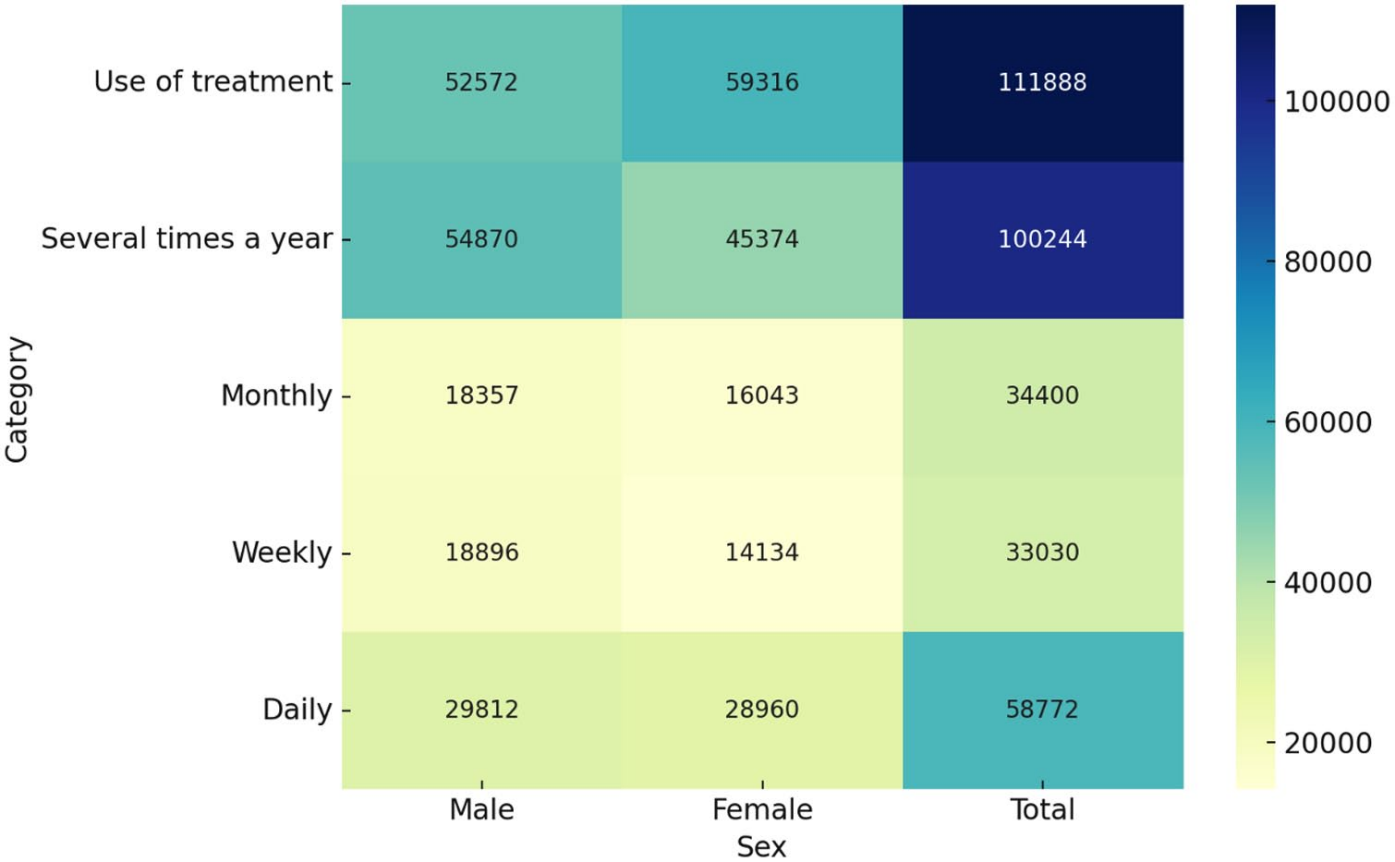
Heatmap of treatment frequency for anxiety and depression among Saudis with disabilities (15+). *Note*. A chi-square test showed a significant association between sex and frequency of treatment use (several-times/year, monthly, weekly, daily), χ²(3) = 415.86, *P* < .001. The effect size was small, Cramer’s *V* = 0.043, indicating statistically detectable but modest differences in frequency patterns by sex.

Figure 5 displays counts from the Disability Survey 2017 for Saudis aged ≥15 years with disabilities, showing (a) medication use for anxiety/depression and (b) self-reported frequency of feeling worried/nervous/anxious or depressed, by sex. Overall, medication use is the most prevalent category (Total = 111 888), with women reporting more medication use than men (59 316 vs 52 572). For symptom frequency, men report higher counts than women across all time frames: several times a year (54 870 vs 45 374), monthly (18 357 vs 16 043), weekly (18 896 vs 14 134), and daily (29 812 vs 28 960). Ordering the magnitudes by total count shows several times a year (100 244) > daily (58,772) > monthly (34,400) > weekly (33,030), indicating that intermittent symptoms are common but a sizeable subgroup experiences daily distress. Taken together, the heatmap suggests a dual pattern: comparatively greater pharmacological treatment among women, alongside more frequent symptom reports among men; given the aggregate nature of the dataset (no diagnosis/severity breakdown), these sex differences should be interpreted cautiously as they may reflect differences in reporting, disability profiles, or care pathways rather than true underlying morbidity alone.

**Figure 6.**
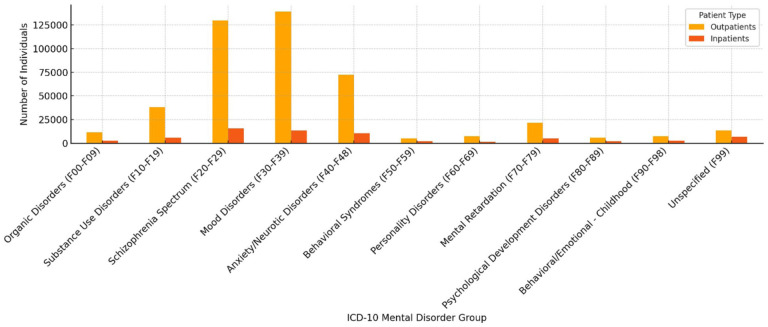
Outpatient and inpatient visits by ICD-10 mental disorder groups in MOH Mental Health Departments (2018). *Note*. A chi-square test indicated a strong association between ICD-10 diagnostic category and treatment setting (outpatient vs inpatient), χ²(10) = 24,837.93, *P* < .001. Effect size was small-to-moderate, Cramer’s *V* = 0.212, reflecting that some groups (eg, F20-F29, F10-F19) are disproportionately represented among inpatients, whereas others (eg, F30-F39, F40-F48) are concentrated in outpatient care. These patterns highlight systemic diagnostic skew, with severe and psychotic conditions dominating inpatient admissions and more common emotional disorders remaining largely within outpatient management.

Figure 6 displays the distribution of outpatient and inpatient visits to mental health departments under the Ministry of Health in Saudi Arabia during 2018, categorized by ICD-10 mental disorder groups. The data reveals that mood disorders (F30-F39) and schizophrenia spectrum disorders (F20-F29) account for the largest volumes of both outpatient (139 323 and 129 902 respectively) and inpatient services (13 706 and 15 723 respectively). Anxiety-related disorders (F40-F48) also contribute substantially to outpatient care (72,484), but less so to inpatient treatment (10,634), indicating that such conditions may more often be managed in outpatient settings. Conversely, less frequent diagnoses like behavioral syndromes (F50-F59) and personality disorders (F60-F69) have significantly lower service usage across both care modalities. These patterns suggest a system that prioritizes service capacity and clinical focus on mood and psychotic disorders, while potentially under-serving developmental, behavioral, and unspecified conditions in both inpatient and outpatient domains.

**Figure 7.**
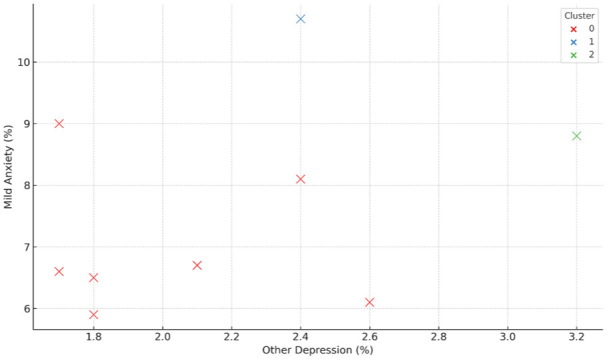
Clustered Mental Health Profiles by Depression and Anxiety Symptoms. *Note*. *K*-means clustering of mild anxiety (%) and other depression (%) across population subgroups. The algorithm identified 3 distinct clusters: Cluster 0 (red) representing lower comorbidity groups, Cluster 1 (blue) showing elevated mild anxiety prevalence, and Cluster 2 (green) highlighting higher co-occurrence of depression and anxiety. *K*-means clustering was applied to move beyond descriptive statistics and uncover latent subgroup patterns not visible in standard prevalence estimates. The elbow method was used to determine the optimal number of clusters, and multiple initializations were tested to ensure robustness.

Figure 7 presents a clustering analysis of depression and anxiety symptom prevalence among the Saudi population aged 15 years and above, using data from the National Health Survey 2024. The visualization maps the relationship between other forms of depression and mild anxiety across age groups and identifies 3 latent mental health profiles through *k*-means clustering. Cluster 0, represented in red, encompasses middle-aged individuals (approximately 30-50 years) characterized by moderate prevalence of both depression and anxiety symptoms, suggesting a balanced emotional health profile. Cluster 1, shown in blue, includes younger adults (15-24 years) who exhibit elevated levels of mild anxiety but comparatively lower depression rates, pointing to early-stage emotional distress possibly linked to developmental or societal transitions. Cluster 2, indicated in green, consists of older adults (primarily those aged 55 and above) and is distinguished by the highest levels of symptom co-occurrence, including a marked rise in depression severity. This clustering highlights demographic heterogeneity in mental health burdens and underscores the importance of stratified, age-specific public health responses—preventive interventions for youth, balanced support systems for middle-aged populations, and intensive mental health care strategies for older adults exhibiting dual symptom escalation.

Table 2 is an integrative synthesis that was developed through analysis of all 8 indicators covering population-level survey data (Indicators 1-6), disability-focused mental health burden (Indicator 7), and Ministry of Health (MOH) clinical service utilization records (Indicator 8). The aim was to evaluate the alignment between self-reported prevalence of depression and anxiety symptoms and the national health system’s diagnostic and service response capabilities. The table is structured around 4 core dimensions of health system performance—coverage, equity, accessibility, and efficiency—plus a fifth axis reflecting diagnostic inclusivity. These domains were selected based on their relevance to evaluating how comprehensively and effectively a mental health system addresses the needs of its population.

**Table 2.** Synthesis of Survey and Clinical Insights on Mental Health in Saudi Arabia.

System dimension	Key evidence (Indicators)	Gap identified	Strategic recommendation
Coverage	Indicators 1-4: National Health Survey shows ≥7% mild anxiety and 2% to 3% depression prevalence across adult groups	Survey-based symptom burden not mirrored in clinical visits (Indicator 8); especially for mild/moderate cases	Implement population-wide screening in schools, workplaces, and PHCs for early detection
Equity	Indicators 5-6: Children aged 11 to 14 report ~4% to 6% mild symptoms; Indicator 7: Disabled population shows intense treatment use	Pediatric and disabled populations are underrepresented in MOH diagnostic records	Expand youth-focused services and integrate disability-mental health services under one platform
Accessibility	Indicator 7: High daily/monthly treatment use among disabled adults; no structured data on access pathways	Access likely reactive and diagnosis-driven; services may not reach non-diagnosed yet symptomatic individuals	Deploy mobile/community MH outreach units, reduce barriers to self-referral
Efficiency	Indicator 8: Over 70% of MOH visits cluster in 3 ICD-10 categories (mood, psychotic, neurotic); early-stage cases under-treated	Over-reliance on psychiatric-level treatment; low outpatient penetration of mild cases	Introduce stepped care models; train PHC providers for front-line mental health care
Diagnostic Inclusivity	Indicators 5-6 versus 8: High prevalence of child symptoms, low service utilization; F80-F98 codes sparsely represented	Under-detection of developmental/behavioral conditions	Establish early diagnostic pathways and psycho-educational assessments in schools

Under coverage, Indicators 1 to 4 reveal that approximately 7.1% of the adult population report mild anxiety symptoms and 2.1% to 2.4% report depressive symptoms. However, Indicator 8 shows that outpatient and inpatient services are predominantly used for mood and psychotic disorders, suggesting that many individuals with mild or moderate conditions are either not engaging with clinical services or are not being diagnosed. This gap underscores the need for upstream screening strategies embedded in non-specialist care environments such as schools and workplaces. For equity, the table highlights that while both children (Indicators 5 and 6) and individuals with disabilities (Indicator 7) exhibit substantial mental health needs—children with up to 6% mild symptoms and disabled adults with heavy treatment burdens—these groups are notably underrepresented in MOH service categories. For instance, few diagnostic entries exist under ICD-10 codes F80 to F98, which relate to developmental and childhood disorders. This discrepancy reflects a service access gap for vulnerable populations, calling for the expansion of youth and disability-focused mental health initiatives. In terms of accessibility, the disability data reveals that a high proportion of individuals are undergoing frequent (daily/monthly) treatment, yet the system offers no direct evidence of community-based or preventative service access points. The inference is that access to care is likely reactive and dependent on formal diagnosis, limiting reach to early-stage or self-identified symptom groups.

The efficiency analysis reveals a clinical system heavily weighted toward late-stage and severe diagnoses. Over 70% of visits in Indicator 8 fall into 3 ICD-10 categories: mood, schizophrenia-spectrum, and anxiety-related disorders. This pattern suggests limited use of stepped-care models, where milder cases could be treated in primary care settings. The imbalance emphasizes the need for a tiered service model where PHC-level providers are trained to handle early interventions. Finally, under diagnostic inclusivity, the sharp disconnect between child symptom prevalence in surveys and the virtual absence of pediatric diagnoses in MOH records suggests structural under-detection of developmental and behavioral mental health conditions. This necessitates the development of formal early diagnostic pathways, particularly within educational and primary pediatric care systems.

To frame the integrative system-level analysis, the study applied the World Health Organization’s Mental Health Action Plan 2013 to 2030 as an analytical framework.^54^ This framework outlines 4 strategic objectives that guide the development of national mental health systems: (1) effective leadership and governance, (2) integrated and responsive service delivery, (3) mental health promotion and prevention, and (4) strengthened information systems and evidence. By mapping the findings from Indicators 1 through 8 to these 4 domains, the analysis identified critical gaps and opportunities in the Saudi mental health ecosystem. Refer to Table 3.

**Table 3.** Mapping Saudi Arabia’s Mental Health System Gaps Against the WHO Mental Health Action Plan 2013 to 2030.

WHO objective	Mapped indicators	Key findings	System gaps identified
1. Effective leadership and governance for mental health	7 (Disability); 5-6 (Children)	Disabled and pediatric populations with high mental health needs are not reflected in coordinated or cross-sector policies.	Weak policy coordination between mental health, education, and disability sectors.
2. Integrated and responsive mental health services in community settings	1-4 (Adults); 8 (Service data)	High prevalence of mild/moderate depression and anxiety is not represented in service usage data; services target severe/chronic cases.	PHC and early intervention layers of care underdeveloped; clinical access limited to severe cases.
3. Promotion and prevention in mental health	5-6 (Children)	Childhood mental health symptoms prevalent, but no documented national prevention strategies or public health interventions.	Lack of school-based mental health promotion or early prevention infrastructure.
4. Strengthened mental health information systems, evidence, and research	1-6 (Surveys); 8 (MOH data)	Survey instruments are robust, but clinical information systems do not link surveillance to service delivery planning.	Fragmentation between epidemiological data and service-level data; poor use of data for planning.

Under Objective 1, governance for mental health remains fragmented, as evident in the lack of coordinated policy action addressing the high symptom burden observed among children and persons with disabilities (Indicators 5-7). Objective 2 emphasizes service responsiveness and community-based care, yet clinical utilization data (Indicator 8) shows a concentration on severe disorders (eg, schizophrenia and mood disorders), with underrepresentation of individuals presenting mild or early-stage symptoms (Indicators 1-4), suggesting underutilization of primary and community care platforms. With respect to Objective 3, survey data reveals widespread subclinical symptoms in children, but there is no corresponding infrastructure for school-based prevention or early interventions. Finally, Objective 4 highlights the need for integrated mental health information systems; although Saudi Arabia maintains strong survey mechanisms, the data is not currently leveraged to inform or calibrate health service delivery models, indicating poor bidirectional integration between epidemiological monitoring and clinical planning.

The use of this framework reveals both systemic blind spots and promising leverage points. Future policy efforts should align surveillance systems with service development, scale up mental health promotion in educational contexts, and embed early screening tools within PHC settings to close identified coverage and equity gaps.

## Discussion

The aim of this study was to investigate the national prevalence, demographic distribution, and healthcare response to depression and anxiety in Saudi Arabia by analyzing integrated data from 4 national-level sources. Six research questions were posed, exploring prevalence patterns across sex and age groups (Q1), childhood mental health presentations (Q2), mental health experiences among individuals with disabilities (Q3), diagnostic distributions in mental health services (Q4), latent population-level symptom profiles (Q5), and systemic gaps between surveillance data and clinical records (Q6). Key findings revealed pronounced sex and age disparities, elevated prevalence among children and persons with disabilities, service reliance on narrow diagnostic groupings, and the emergence of symptom clusters that elude traditional categorization.

Our findings underscore a profound disconnect between mental health surveillance and service delivery in Saudi Arabia. While survey data revealed high prevalence rates of depression and anxiety—especially among young females—administrative records demonstrated a concentration of clinical attention on psychotic and bipolar disorders, with depression and anxiety comprising a smaller fraction of inpatient and outpatient diagnoses. This divergence aligns with concerns raised in the literature about the underrepresentation of common mental disorders in clinical records due to systemic diagnostic practices and help-seeking barriers.^27,35^ The disparity also resonates with the WHO Mental Health Action Plan’s emphasis on aligning diagnostic recognition with true population-level needs.^58^

Particularly vulnerable subpopulations emerged from the data, including children aged 11 to 14 and individuals with disabilities. Our heatmap analyses revealed substantial early-onset mental health symptoms, especially among adolescent girls—a finding that parallels recent studies documenting high distress levels among Saudi adolescents.^30^ Likewise, data from the Disability Survey revealed significant symptom frequencies and treatment gaps among people with disabilities, reinforcing global calls for intersectional mental health strategies that account for compounded vulnerabilities.^59^ These insights highlight the need for a more inclusive mental health infrastructure that anticipates and addresses the intersecting needs of these subgroups.

The systemic narrowness of diagnostic classification in Saudi mental health services became particularly evident in our examination of ICD-10 codes. The majority of clinical encounters were associated with schizophrenia and mood disorders, while anxiety and related conditions were underrepresented. This skew may reflect a combination of cultural stigma, limited diagnostic training, and institutional prioritization of severe mental illness over common but disabling conditions.^60^ Furthermore, our cluster analysis revealed that symptom profiles in the general population often do not fit neatly into existing categorical diagnoses. This finding supports a growing international discourse advocating for dimensional models of mental health, such as those proposed in the ICD-11 and reflected in WHO’s ICF framework.^19,22^

These findings must also be interpreted within the broader context of social determinants of mental health. In Saudi Arabia, women, youth, and persons with disabilities face structural vulnerabilities compounded by stigma and limited access to tailored services. Barriers to mental health care include shortages of specialized professionals, underdeveloped community-based services, uneven geographic distribution of providers, and persistent cultural stigma surrounding psychiatric treatment.^29,30^ Addressing these barriers is critical for bridging the observed gap between high community prevalence and low service utilization.

Finally, the converging evidence from national surveys and administrative records points toward opportunities for systemic reform. Integrating functional assessment tools from the ICF, adopting earlier screening interventions in primary care, and updating diagnostic training in line with ICD-11 could help bridge the current surveillance–service divide. Our study also contributes to the realization of global health priorities outlined in the United Nations Sustainable Development Goals, particularly SDG 3.4, which advocates reducing premature mortality from non-communicable diseases, including mental health conditions, through prevention and treatment.^44^ By aligning Saudi Arabia’s mental health strategies with these global frameworks, policymakers can promote more responsive and equitable care pathways.

## Implications for Health Policy

The findings of this study offer compelling evidence for urgent policy reforms in mental health planning and service provision in Saudi Arabia. Foremost among these is the need to recalibrate national mental health priorities toward more inclusive diagnostic recognition of depression and anxiety, particularly among children, women, and persons with disabilities. Strengthening early screening protocols in primary healthcare settings, informed by the ICD-11 and ICF frameworks, can facilitate earlier detection and personalized care pathways. Workforce training in dimensional diagnostic approaches and the deployment of culturally adapted assessment tools should be central to such reforms. Furthermore, the high prevalence rates identified in underserved groups demand the expansion of decentralized community mental health services, moving beyond hospital-centric models. These recommendations align with the WHO Mental Health Action Plan^58^ and advance the Kingdom’s progress toward Sustainable Development Goal 3.4, which calls for a reduction in non-communicable diseases—including mental illness—through prevention, treatment, and promotion of mental well-being.^44^ The integration of these strategies within Saudi Arabia’s Vision 2030 health transformation framework could enhance the responsiveness, accessibility, and equity of the nation’s mental health system.

Beyond identifying gaps in surveillance and service alignment, our findings carry important implications for Saudi-specific policy. While integrating functional assessment tools such as the ICF and expanding early screening within primary care are essential, their effectiveness depends on addressing cultural stigma and low mental health literacy. These challenges are widely documented across GCC countries, where stigma, limited knowledge, and negative attitudes toward mental illness remain major barriers to care—even among healthcare professionals.^61^ Qualitative studies in the region further highlight that personal, familial, religious, and systemic factors strongly influence help-seeking decisions, underscoring the need for culturally sensitive approaches.^62^ Ensuring confidentiality in close-knit communities and engaging religious and community leaders will be crucial to reducing stigma and improving uptake of services.

At the regional level, other Gulf countries have begun implementing strategies that could inform Saudi reforms. Under Saudi Vision 2030, mental health has been positioned as a core component of healthcare transformation, with initiatives promoting digital health, early detection, and service integration.^63^ Similarly, regional innovations such as telepsychiatry, mobile outreach, and public awareness campaigns have shown promise in improving accessibility and reducing stigma.^64^ Adapting these approaches to the Saudi context—particularly through integration into primary care, schools, and workplaces—could enhance equity and inclusivity while aligning with ethical and cultural norms. By situating reforms within Saudi Arabia’s cultural, social, and religious context, policy makers can ensure that interventions are both effective and acceptable, fostering sustainable improvements in mental health care delivery.

## Strengths and Limitations

This study presents a unique contribution by triangulating multiple national datasets to generate a multidimensional portrait of depression and anxiety within Saudi Arabia. The integration of prevalence data from health surveys, administrative records from mental health services, and subpopulation-specific data for children and persons with disabilities provides a rare cross-sectional and systemic perspective. This methodological breadth allows for a more nuanced interpretation of mental health patterns across the life course and service continuum. Moreover, the application of the WHO framework, alongside advanced clustering techniques, enabled identification of latent symptom profiles that transcend conventional diagnostic boundaries, adding sophistication to both the analytic approach and the conceptual framing.

However, several limitations must be acknowledged. First, the reliance on self-reported data in national surveys may be subject to response bias, particularly given the stigma surrounding mental illness in Saudi culture. Second, the administrative data from the Ministry of Health reflect only those who accessed formal care, thus underrepresenting untreated or undiagnosed cases. Third, cross-sectional data limit the ability to infer causal relationships or temporal dynamics of mental health trends. Finally, although our study incorporated large-scale national datasets, the absence of certain variables—such as socioeconomic status, medication adherence, or comorbidity profiles—constrains the depth of analysis. Further, a key limitation of this study is the temporal gap between the population survey datasets (2024) and the Ministry of Health mental health service dataset (2018). This mismatch may limit direct comparability between community prevalence and service utilization. However, the 2018 dataset represents the most recent nationally available record of mental health service use in Saudi Arabia and was therefore included to provide critical insight into diagnostic and treatment patterns otherwise unavailable through survey data. Another limitation is the absence of key socioeconomic and regional variables, such as income, education, occupation, and geographic location. The lack of these determinants constrains deeper analysis of structural inequalities in mental health within the Saudi population. Despite these limitations, the study offers valuable insights that may inform both future research and strategic policy interventions. One more limitation is that because this study relied on secondary data, we were not able to access the raw instruments or independently verify their psychometric properties. According to the General Authority for Statistics (GASTAT), all survey tools underwent validation prior to administration; however, the precise psychometric parameters are not publicly reported. Given that these instruments were implemented by a national authority, we assumed that appropriate validation procedures were conducted to ensure measurement quality.

## Conclusion

This study examined the prevalence, demographic distribution, and service utilization patterns related to depression and anxiety in Saudi Arabia, drawing on a triangulated analysis of 4 national-level data sources. The findings reveal notable disparities in symptom severity and prevalence by sex and age, with particularly high burdens among adolescents, women, and individuals with disabilities. Service records further demonstrated a narrow diagnostic concentration within clinical settings, often not reflective of the broader population-level mental health burden. Through multivariate and clustering techniques, distinct latent symptom profiles were identified, suggesting that existing diagnostic models may inadequately capture comorbid or atypical presentations of mental distress.

Compared to existing Saudi studies that have primarily focused on single populations such as university students, healthcare workers, or regional samples, this study advances knowledge by synthesizing multiple national datasets and linking community-level prevalence with health system utilization. This integrative approach reveals systemic mismatches between population needs and clinical focus, highlighting gaps that would not be visible in isolated studies.

These insights are especially relevant to Saudi Arabia’s Vision 2030 Health Sector Transformation Program, which prioritizes preventive care, equitable access, and mental health integration into primary healthcare. Our findings support the expansion of school- and workplace-based screening programs, the establishment of early diagnostic pathways for youth and persons with disabilities, and the development of community mental health services that reduce reliance on hospital-based care. Policy reforms should also address stigma reduction, workforce shortages, and the implementation of stepped-care models to ensure that mild and moderate cases are managed effectively at the primary care level.

Incorporating these recommendations into national strategy offers a promising path toward a more inclusive, responsive, and data-informed mental healthcare system. By aligning evidence-based reforms with Vision 2030 goals and the United Nations Sustainable Development Targets, Saudi Arabia has an opportunity to set a regional benchmark for transforming mental health policy and practice in the Middle East.^65^

## Supplemental Material

sj-docx-2-inq-10.1177_00469580251382027 – Supplemental material for Depression and Anxiety in the Saudi Population: Epidemiological Profiles from Health Surveys and Mental Health Services Supplemental material, sj-docx-2-inq-10.1177_00469580251382027 for Depression and Anxiety in the Saudi Population: Epidemiological Profiles from Health Surveys and Mental Health Services by Ahmed Yahya Almakrob and Ahmed Alduais in INQUIRY: The Journal of Health Care Organization, Provision, and Financing

sj-pdf-1-inq-10.1177_00469580251382027 – Supplemental material for Depression and Anxiety in the Saudi Population: Epidemiological Profiles from Health Surveys and Mental Health Services Supplemental material, sj-pdf-1-inq-10.1177_00469580251382027 for Depression and Anxiety in the Saudi Population: Epidemiological Profiles from Health Surveys and Mental Health Services by Ahmed Yahya Almakrob and Ahmed Alduais in INQUIRY: The Journal of Health Care Organization, Provision, and Financing

## References

[1] Verma P Srivastava R Srivastava S . Identification of unipolar depression using boosting algorithms. In: Pandey R Maurya P Chiong R , eds. Data Modelling and Analytics for the Internet of Medical Things. CRC Press; 2023:126-141.

[2] Kohn R . Epidemiology of common mental disorders. In: H Budde, M Wegner, eds. The Exercise Effect on Mental Health: Neurobiological Mechanisms. Taylor & Francis; 2018:3-29.

[3] Torzsa P Szeifert L Dunai K Kalabay L Novák M . [Diagnosis and therapy of depression in family practice]. Orv Hetil. 2009;150(36):1684-1693. doi:10.1556/OH.2009.28675 19709983

[4] Lubecka B Lubecki M Pudlo R . Epidemiology of anxiety and depressive disorders. Psychiatry. 2022;19(1):66-77. doi:10.5603/PSYCH.a2021.0034

[5] Wang J Guan X Tao N . GBD: incidence rates and prevalence of anxiety disorders, depression and schizophrenia in countries with different SDI levels, 1990-2021. Front Public Health. 2025;13:1556981. doi:10.3389/fpubh.2025.1556981 40453495 PMC12124139

[6] Liu J Ning W Zhang N Zhu B Mao Y . Estimation of the global disease burden of depression and anxiety between 1990 and 2044: an analysis of the Global Burden of Disease Study 2019. Healthcare. 2024;12(17):1721. doi:10.3390/healthcare12171721 39273745 PMC11395616

[7] Gaudlitz K von Lindenberger BL Zschucke E Ströhle A . Mechanisms underlying the relationship between physical activity and anxiety: human data. In: P Ekkekakis, e﻿d. Routledge Handbook of Physical Activity and Mental Health. Routledge; 2013.

[8] Baxter AJ Vos T Scott KM , et al The regional distribution of anxiety disorders: implications for the Global Burden of Disease Study, 2010. Int J Methods Psychiatr Res. 2014;23(4):422-438. doi:10.1002/mpr.1444 25048296 PMC6878273

[9] Yuan LL Lu L Wang XH , et al Prevalence and predictors of anxiety and depressive symptoms among international medical students in China during COVID-19 pandemic. Front Psychiatry. 2021;12:761964. doi:10.3389/fpsyt.2021.761964 34803770 PMC8599347

[10] Sahoo S Khess CR . Prevalence of depression, anxiety, and stress among young male adults in India: a dimensional and categorical diagnoses-based study. J Nerv Ment Dis. 2010;198(12):901-904. doi:10.1097/NMD.0b013e3181fe75dc 21135643

[11] Rai D Zitko P Jones K Lynch J Araya R . Country- and individual-level socioeconomic determinants of depression: multilevel cross-national comparison. Br J Psychiatry. 2013;202(3):195-203. doi:10.1192/bjp.bp.112.112482 23349294

[12] Ilgün G . What are the socioeconomic determinants of mental disorders? Perspect Psychiatr Care. 2022;58(4):2881-2887. doi:10.1111/ppc.13136 35780329

[13] Heim E Wegmann I Maercker A . Cultural values and the prevalence of mental disorders in 25 countries: a secondary data analysis. Soc Sci Med. 2017;189:96-104. doi:10.1016/j.socscimed.2017.07.024 28793240

[14] Getie A Ayalneh M Bimerew M . Global prevalence and determinant factors of pain, depression, and anxiety among cancer patients: an umbrella review of systematic reviews and meta-analyses. BMC Psychiatry. 2025;25(1):156. doi:10.1186/s12888-025-06599-5 39972435 PMC11841195

[15] Cuijpers P Eylem O Karyotaki E Zhou X Sijbrandij M . Psychotherapy for depression and anxiety in low- and middle-income countries. In: DJ Stein, JK Bass, SG Hofmann, e﻿ds. Global Mental Health and Psychotherapy: Adapting Psychotherapy for Low- and Middle-Income Countries. Elsevier; 2019:173-192.

[16] Maina G Mauri M Rossi A . Anxiety and depression. J Psychopathol. 2016;22(1):28-38.

[17] van Balkom AJ Gabriëls L van den Heuvel OA . [Anxiety, obsessive-compulsive disorder and trauma in the DSM-5]. Tijdschr Psychiatr. 2014;56(3):177-181. 24643827

[18] Park SC Kim YK . Anxiety disorders in the DSM-5: Changes, controversies, and future directions. In: Kim YK , ed. Anxiety Disorders. Vol. 1191. Springer, 2020:1-12. doi:10.1007/978-981-32-9705-0_12 32002930

[19] Ziebold C Mari JJ Goldberg DP , et al Diagnostic consequences of a new category of anxious depression and a reduced duration requirement for anxiety symptoms in the ICD-11 PHC. J Affect Disord. 2019;245:120-125. doi:10.1016/j.jad.2018.10.082 30368071

[20] de Dios C Goikolea JM Colom F Moreno C Vieta E . Bipolar disorders in the new DSM-5 and ICD-11 classifications. Rev Psiquiatr Salud Ment. 2014;7(4):179-185. doi:10.1016/j.rpsm.2014.07.005 25450512

[21] Ertle A Lüken U . Anxiety or fear-related disorders in the ICD-11. Nervenheilkunde. 2024;43(4):203-208. doi:10.1055/a-2216-7172

[22] Kogan CS Stein DJ Maj M First MB Emmelkamp PM Reed GM . The classification of anxiety and fear-related disorders in the ICD-11. Depress Anxiety. 2016;33(12):1141-1154. doi:10.1002/da.22530 27411108

[23] Shevlin M Hyland P Vallières F , et al A comparison of DSM-5 and ICD-11 PTSD prevalence, comorbidity and disability: an analysis of the Ukrainian internally displaced Person’s Mental Health Survey. Acta Psychiatr Scand. 2018;137(2):138-147. doi:10.1111/acps.12840 29210054

[24] Petrova NN Savickaya KS . The diagnosis and therapy of comorbid anxiety and depression disorders in clinical practice. Bekhterev Rev Psychiatry Med Psychol. 2021;128(1):102-112. doi:10.31363/2313-7053-2021-1-102-112

[bibr25-00469580251382027] Clark LA Watson D . Distress and fear disorders: an alternative empirically based taxonomy of the “mood” and “anxiety” disorders. Br J Psychiatry. 2006;189:481-483. doi:10.1192/bjp.bp.106.03825 17139030

[26] Stein DJ Craske MG Friedman MJ Phillips KA . Meta-structure issues for the DSM-5: how do anxiety disorders, obsessive-compulsive and related disorders, post-traumatic disorders, and dissociative disorders fit together? Curr Psychiatry Rep. 2011;13(4):248-250. doi:10.1007/s11920-011-0207-1 21603904

[27] Alhabeeb AA Al-Duraihem RA Alasmary S Alkhamaali Z Althumiri NA BinDhim NF . National screening for anxiety and depression in Saudi Arabia 2022. Front Public Health. 2023;11:1213851. doi:10.3389/fpubh.2023.1213851 37441650 PMC10333514

[28] Alamri HS Algarni A Shehata SF , et al Prevalence of depression, anxiety, and stress among the general population in Saudi Arabia during COVID-19 pandemic. Int J Environ Res Public Health. 2020;17(24):9183. doi:10.3390/ijerph17249183 33316900 PMC7764434

[29] Almalki AH Alzahrani MS Alshehri FS , et al The psychological impact of COVID-19 on healthcare workers in Saudi Arabia: a year later into the pandemic. Front Psychiatry. 2021;12:797545. doi:10.3389/fpsyt.2021.797545 34975592 PMC8718633

[30] Malebari AM Alamoudi SO Al-Alawi TI Alkhateeb AA Albuqayli AS Alothmany HN . Prevalence of depression and anxiety among university students in Jeddah, Saudi Arabia: exploring sociodemographic and associated factors. Front Public Health. 2024;12:1441695. doi:10.3389/fpubh.2024.1441695 39726655 PMC11670204

[31] Alfakeh SA Alghamdi KM Hilal RM Abdelmoaty MM . Rates of depression, anxiety, and stress in King Abdulaziz University freshmen, Jeddah, Saudi Arabia: a cross-sectional study. J Contemp Med Sci. 2021;7(2):116-121.

[32] Alfaqih AM Alqassim AY Hakami MH , et al The impact of physical activity on depression, anxiety, and stress during pregnancy in Saudi Arabia: A cross-sectional study. Medicinar. 2024;60(8):1263. doi:10.3390/medicina60081263 PMC11355941 39202544

[33] Al-Zahrani R Bashihab R Ahmed AE Alkhodair R Al-Khateeb S . The prevalence of psychological impact on caregivers of hospitalized patients: the forgotten part of the equation. Qatar Med J. 2015;2015(1):3. doi:10.5339/qmj.2015.3 26535171 PMC4614325

[34] AlAteeq D AlNujaim SM AlGharbi AH . Prevalence of depression and anxiety among working women in Saudi Arabia: psychosocial and perinatal correlates. BMC Womens Health. 2025;25(1):228. doi:10.1186/s12905-025-03776-2 40375291 PMC12079880

[35] Al-Shehri SZ Sabra AA Taha AZ Khamis AH Hafez AS . Depression and anxiety among males attending primary health care centers, eastern Saudi Arabia: prevalence and predictors. Life Sci J. 2012;9(3):128-133.

[36] Almalki M Fitzgerald G Clark M . Health care system in Saudi Arabia: an overview. East Mediterr Health J. 2011;17(10):784-793. doi:10.26719/2011.17.10.784 22256414

[37] Rahman R Salam MA . Policy discourses: shifting the burden of healthcare from the state to the market in the Kingdom of Saudi Arabia. Inquiry. 2021;58:469580211017655. doi:10.1177/00469580211017655 PMC8142522 34014129

[38] Almasabi MH . An overview of health system in Saudi Arabia. Res J Med Sci. 2013;7(3):70-74.

[39] Alkhamis A Hassan A Cosgrove P . Financing healthcare in Gulf Cooperation Council countries: a focus on Saudi Arabia. Int J Health Plann Manage. 2014;29(1):e64-e82. doi:10.1002/hpm.2213 PMC4260721 23996348

[40] Al-Anezi FM . Challenges of healthcare systems in Saudi Arabia to delivering Vision 2030: an empirical study from healthcare workers’ perspectives. J Healthc Leadersh. 2025;17:173-187. doi:10.2147/JHL.S516159 40357423 PMC12067643

[41] Carlisle J . Mental health law in Saudi Arabia. BJPsych Int. 2018;15(1):17-19. doi:10.1192/bji.2017.10 29953138 PMC6020922

[42] Almadani AH Altheyab ES Alkheraiji MA , et al Perceptions and attitudes of mental health professionals toward the mental health care law in Saudi Arabia. Healthcare. 2023;11(20):2784. doi:10.3390/healthcare11202784 37893858 PMC10606621

[43] Alblowi EA Shujaa MA Alonazi WB . Measuring performance of rural mental healthcare services in Saudi Arabia. Psychol Res Behav Manag. 2023;16:3895-3905. doi:10.2147/PRBM.S420662 37817911 PMC10561611

[44] United Nations. Sustainable Development Goals. United Nations Department of Economic and Social Affairs. Published 2024. Accessed August 22, 2025. https://sdgs.un.org/goals

[45] General Authority for Statistics. Health Status Statistics 2024. General Authority for Statistics; 2024. Accessed August 22, 2025. https://www.stats.gov.sa/en

[46] General Authority for Statistics. Methodology and Quality Report of Health Condition Statistics Publication. General Authority for Statistics; 2024. Accessed August 22, 2025. https://www.stats.gov.sa/en

[47] General Authority for Statistics. Disability Survey 2017. General Authority for Statistics; 2017. Accessed August 22, 2025. https://www.stats.gov.sa/en

[48] Ministry of Health (Saudi Arabia). Mental Health Services and Programs. Ministry of Health; 2024. Accessed August 22, 2025. https://www.moh.gov.sa/EN/Pages/default.aspx

[49] World Health Organization. Mental Health Action Plan 2013–2020. World Health Organization; 2013. Accessed August 22, 2025. https://www.who.int/publications/i/item/9789241506021

[50] Bickenbach J Cieza A Sabariego C . Disability and public health. Int J Environ Res Public Health. 2016;13(1):123. doi:10.3390/ijerph13010123 PMC4730457 26703704

[51] Thornicroft G Tansella M . The balanced care model for global mental health. Psychol Med. 2013;43(4):849-863. doi:10.1017/S0033291712001420 22785067

[52] von Elm E Altman DG Egger M , et al The strengthening the reporting of observational studies in Epidemiology (STROBE) statement: guidelines for reporting observational studies. Ann Intern Med. 2007;147(8):573-577. doi:10.7326/0003-4819-147-8-200710160-00010 17938396

[bibr53-00469580251382027] von Elm E Altman DG Egger M , et al The strengthening the reporting of observational studies in Epidemiology (STROBE) statement: guidelines for reporting observational studies. PLoS Med. 2007;4(10):e296. doi:10.1371/journal.pmed.0040296 PMC2020495 17941714

[54] von Elm E Altman DG Egger M , et al Strengthening the reporting of observational studies in Epidemiology (STROBE) statement: guidelines for reporting observational studies. BMJ. 2007;335(7624):806-808. doi:10.1136/bmj.39335.541782.AD 17947786 PMC2034723

[55] Washington Group on Disability Statistics. Washington Group Extended Set on Functioning (WG-ES). Washington Group on Disability Statistics. Published n.d. Accessed August 22, 2025. https://www.washingtongroup-disability.com/question-sets/wg-extended-set-on-functioning-wg-es/

[56] Saudi Open Data Portal. Open data repository of the Kingdom of Saudi Arabia. Published 2024. Accessed August 22, 2025. https://open.data.gov.sa/en/home

[57] World Medical Association. World Medical Association Declaration of Helsinki: ethical principles for medical research involving human subjects. JAMA. 2013;310(20):2191-2194. doi:10.1001/jama.2013.281053 24141714

[58] World Health Organization. Comprehensive Mental Health Action Plan 2013–2030. World Health Organization; 2021.

[59] World Health Organization. World Mental Health Report: Transforming Mental Health for All. World Health Organization; 2022.

[60] Alzahrani F Alshahrani NZ Abu Sabah A Zarbah A Abu Sabah S Mamun MA . Prevalence and factors associated with mental health problems in Saudi general population during the coronavirus disease 2019 pandemic: a systematic review and meta-analysis. Psych J. 2022;11(1):18-29. doi:10.1002/pchj.516 34986503

[61] Elyamani R Naja S Al-Dahshan A Hamoud H Bougmiza MI Alkubaisi N . Mental health literacy in Arab states of the Gulf Cooperation Council: a systematic review. PLoS One. 2021;16(1):e0245156. doi:10.1371/journal.pone.0245156 PMC7790272 33411793

[62] Alageel S Alzahrani N Alsaidi A Alqahtani M Aldossary A . Factors influencing decisions to seek mental healthcare in the Gulf Cooperation Council: a qualitative study. BMC Public Health. 2025;25(1):112607. doi:10.1186/s12889-025-21607-9 PMC12004681 40240998

[63] Sharma S Basumatary P Van Steendam F Bacha E . Strategies to Transform Mental Healthcare in the GCC. Oliver Wyman; 2025. Accessed August 22, 2025. https://www.oliverwyman.com/our-expertise/insights/2025/apr/transform-mental-healthcare-gcc.html

[64] Alanazi TNM McKenna L Buck M . The nature and availability of mental health services in Arab Gulf countries: a scoping review. Saudi J Health Syst Res. 2023;3(1-4):10-34. doi:10.1159/000531699

[65] Saudi Vision 2030. Health Sector Transformation Program. Government of Saudi Arabia; 2024. Accessed August 22, 2025. https://www.vision2030.gov.sa

