# Supplementary material for: Depression and Anxiety in the Saudi Population: Epidemiological Profiles from Health Surveys and Mental Health Services
Source: Inquiry. 2025 Oct 16;62:00469580251382027. doi: 10.1177/00469580251382027 (PMC12536202; doi:10.1177/00469580251382027)
Supplement: sj-docx-2-inq-10.1177_00469580251382027 – Supplemental material for Depression and Anxiety in the Saudi Population: Epidemiological Profiles from Health Surveys and Mental Health Services [file sj-docx-2-inq-10.1177_00469580251382027.docx]

# Supplementary file 2.

# STROBE Statement—Checklist for Cross-Sectional Studies (Tailored to Manuscript)

Note: This checklist follows STROBE (Strengthening the Reporting of Observational Studies in Epidemiology) for cross-sectional studies. It is pre-filled using your current manuscript structure. Because Word pagination is dynamic, page numbers should be confirmed after final layout. Use the blank 'Page' column to fill the exact page in your final file.

| Item | STROBE Recommendation | Where in Manuscript (section/paragraph) | Page |
| --- | --- | --- | --- |
| 1a | Indicate study design in title/abstract | Abstract – Methods; Title | 1 |
| 1b | Provide informative, balanced abstract | Abstract (all subheadings) | 1 |
| 2 | Explain scientific background/rationale | Introduction – Prevalence internationally; DSM/ICD/ICF sections | 1-3 |
| 3 | State specific objectives/hypotheses | Purpose of the Present Study; Research Questions (Q1–Q6) | 3-4 |
| 4 | Present key elements of study design early | Methods – Design | 5-6 |
| 5 | Describe setting, locations, dates | Methods – Sample; Procedure – Data Collection | 5-6 |
| 6a | Eligibility criteria; sources/methods of selection | Methods – Sample | 5-6 |
| 7 | Define outcomes/exposures/confounders; diagnostic criteria | Methods – Measures; WG‑ES and ICD-10 groups | 6-7 |
| 8* | Data sources/measurement details for each variable | Methods – Measures; Triangulation approach | 6-7 |
| 9 | Describe efforts to address bias | Methods – Triangulation; Strengths and Limitations | 7-8 |
| 10 | Explain study size determination | Methods – Measures (All available cases) | 6-8 |
| 11 | Handling of quantitative variables/groupings | Methods – Measures; Data Analysis | 7-8 |
| 12a | Describe all statistical methods | Methods – Data Analysis | 7-8 |
| 12b | Methods for subgroups/interactions | Methods – Data Analysis; Results – subgroup figures | 6-8 |
| 12c | Explain how missing data were addressed | Methods – Measures (no missing entries) | 6-8 |
| 12d | Analytical methods accounting for sampling strategy | Methods – Sample; Data Analysis | 7-8 |
| 12e | Describe any sensitivity analyses | Methods – Data Analysis | NA |
| 13* | Report participant numbers at each stage; reasons for non-participation; flow diagram | Results (figures/tables) | 8-17 |
| 14a* | Give characteristics of study participants | Results – (could add a small table of N by age/sex) | NA |
| 14b* | Indicate number of participants with missing data per variable | Methods – Measures | NA |
| 15* | Report numbers of outcome events or summary measures | Results – Figures 1–6; narrative | 8-17 |
| 16 | Main results with estimates/precision; confounder adjustment | Results | 8-17 |
| 17 | Other analyses (subgroups, interactions, sensitivity) | Results – Cluster analysis; heatmaps | 8-17 |
| 18 | Summarise key results wrt objectives | Discussion – opening paragraph | 17-18 |
| 19 | Discuss limitations (bias, imprecision, magnitude/direction) | Strengths and Limitations | 19 |
| 20 | Provide cautious interpretation considering context | Discussion – interpretation paragraphs | 17-19 |
| 21 | Discuss generalisability (external validity) | Discussion – policy/vision linkage; Implications | 18-19 |
| 22 | Funding source/role of funders | End matter (if applicable) | 20 |

*Items marked with an asterisk (*) request information separately for exposed/unexposed groups; for this descriptive multi-source study, report by key strata (age, sex, disability) as you have done.

# STROBE Compliance

## 1) Design (Methods) – STROBE Statement

This study is reported in accordance with the STROBE (Strengthening the Reporting of Observational Studies in Epidemiology) checklist for cross‑sectional studies. The observational design was cross‑sectional and multi‑source, leveraging nationally representative survey and administrative datasets.

## 2) Participants (Methods – Sample) – Explicit Inclusion Criteria

Inclusion criteria were defined by the sampling frames of the original national datasets. We included: (i) all respondents aged ≥15 years in the National Health Survey 2024 who completed the mental‑health modules; (ii) all children aged 11–14 years in the Woman and Child Health Survey 2024 with completed emotional health items; (iii) all individuals aged ≥15 years in the Disability Survey 2017 who reported ≥1 disability and completed the affect module; and (iv) all outpatient and inpatient encounters recorded in Ministry of Health mental health departments during 2018. No additional exclusion criteria were applied beyond those inherent to the source datasets.

## 3) Bias – One‑sentence Addition (Methods and/or Limitations)

Potential information bias due to self‑report was mitigated by using standardized instruments and consistent field procedures; selection bias was minimized by the stratified multistage probability sampling used by GASTAT.

## 4) Sampling Strategy / Weights (Statistical Methods 12d)

If survey weights are available from GASTAT, add: 'All prevalence estimates were computed using survey weights supplied by GASTAT to account for the complex sampling design.' If weights are unavailable, add: 'Survey weighting variables were not available in the public‑use files; unweighted estimates are presented with stratification by age and sex.'

## 5) Sensitivity/Robustness (12e – optional)

As an optional robustness check, report that clustering results were stable across multiple random initializations and k in {2,3,4} with the elbow method supporting k=3.

## 6) Generalisability (21)

These findings are generalisable to residents of Saudi Arabia living in private households covered by the national sampling frames; clinical utilisation patterns may differ in non‑MOH sectors or non‑household populations (e.g., institutionalised, remote, or transient groups).

## 7) Funding (22)

Add a Funding/Acknowledgments statement specifying financial support and the role of funders, or state 'No specific funding was received.'

## 8) Psychometrics / Instrument Validation (Limitations)

Because this study relied on secondary data, we did not access raw instruments or independently verify psychometric properties. According to GASTAT, all survey tools underwent validation before implementation; precise psychometric parameters are not publicly reported. Given implementation by a national authority, we assumed appropriate validation procedures ensuring measurement quality.
